# Incidence of cardioembolic stroke related to atrial fibrillation in Joinville, Brazil

**DOI:** 10.1055/s-0043-1767821

**Published:** 2023-05-09

**Authors:** Henrique Diegolli, Raddib Eduardo Noleto da Nobrega Oliveira, Caroline Figueiredo da Silva, Gustavo Figueiredo da Silva, Felipe Fanine de Souza, Felipe Reinert Avilla Machado, Marcelo Pitombeira de Lacerda

**Affiliations:** 1 Hospital Municipal São José, Departamento de Medicina Interna, Serviço de Neurologia, Joinville SC, Brazil.; 2 Universidade da Região de Joinville, Departamento de Medicina, Joinville SC, Brazil.; 3 Hospital Municipal São José, Departamento de Medicina Interna, Serviço de Hematologia, Joinville SC, Brazil.

**Keywords:** Atrial Fibrillation, Ischemic Stroke, Embolic Stroke, Cohort Studies, Fibrilação Atrial, AVC Isquêmico, AVC Embólico, Estudos de Coorte

## Abstract

**Background** Atrial fibrillation (AF) is an important cause of cardioembolic stroke, and population aging has increased its prevalence.

**Objective** To evaluate the incidence of cardioembolic stroke caused by AF in the city of Joinville, Brazil, as well as previous diagnoses and use of medication.

**Methods** Between 2017 and 2020 we extracted data from the population-based Joinville Stroke Registry. Demographic characteristics, diagnosis of AF, and patterns of medication use were collected, and the Trial of ORG 10172 in Acute Stroke Treatment (TOAST) system was used to classify the etiology.

**Results** There were 3,303 cases of ischemic stroke, 593 of which were cardioembolic, and 360 had AF. Of the patients with AF, 258 (71.6%) had a previous diagnosis of the disease, and 102 (28.3%) were newly diagnosed after the stroke. Among patients with a previously-diagnosed AF, 170 (47.2%) were using anticoagulants, and 88 (24.4%) were using other medications.

**Conclusion** During the analyzed period, ischemic stroke caused by AF was a significant burden on the population of Joinville, and a considerable number of patients had undiagnosed or untreated AF.

## Resumo

**Antecedentes** A fibrilação atrial (FA) é uma importante causa de acidente vascular cebebral (AVC) cardioembólico, e o envelhecimento populacional aumentou a sua prevalência.

**Objetivo** Avaliar a incidência de AVC cardioembólico causado por FA em Joinville, além dos diagnósticos prévios e do uso de medicamentos.

**Métodos** Entre 2017 e 2020, foram extraídos dados do registro de base populacional de AVC de Joinville. Características demográficas, diagnóstico de FA e padrões de uso de medicamentos foram coletados, e o sistema Trial of ORG 10172 in Acute Stroke Treatment (TOAST) foi utilizado para classificar a etiologia.

**Resultados** Houve 3.303 casos de AVC isquêmico, sendo 593 cardioembólicos e 360 com FA. Dos pacientes com FA, 258 (71,6%) tinham diagnóstico prévio da doença, e 102 (28,3%) foram recém-diagnosticados após o AVC. Entre os pacientes com FA previamente diagnosticada, 170 (47,2%) estavam em uso de anticoagulante, e 88 (24,4%), em uso de outra medicação.

**Conclusão** Durante o período analisado, o AVC isquêmico causado por FA foi um ônus significativo para a população de Joinville, e um número considerável de pacientes apresentava FA não diagnosticada ou não tratada.

## INTRODUCTION

Atrial fibrillation (AF) is responsible for a high rate of cardio- and cerebrovascular morbidity and mortality, and it is among the most prevalent heart rhythm disorders. Among the causes of cardioembolic stroke, the most important is AF, an independent risk factor for stroke, which increases its risk between 2- to 5-fold. The prevalence increases with age, and among patients > 80 years old, it is of almost 10%. 1

It is estimated that cardioembolic stroke accounts for 15% to 20% of all ischemic strokes, and may be more severe than other subtypes, such as those caused by the occlusion of small vessels. 1 Data from the JOINVASC population-based cohort 2 have shown that the cardioembolic subtype had the highest severity when compared with other identified stroke etiologies. One month after a stroke, 40.3% of the patients with the cardioembolic subtype were severely disabled compared with 28.3% of patients with large-artery atherosclerosis and 7.7% of patients with small-artery occlusion. Only 39.4% of the patients who had a cardioembolic stroke were still alive 5 years later, compared with 53.0% of patients with large-artery atherosclerosis and 69.9% of patients with small-artery occlusion. 2

Stroke caused by an AF has been shown to be more severe since the Framingham study, 3 which found that AF nearly doubled the risk of death within 30 days, and recurrences were more common than in other subtypes. Strokes related to AF are particularly severe and have the highest rates of mortality and disability, with a 50% chance of dying within a year compared with 27% for strokes unrelated to AF. 4 5 The long-term outcomes of AF-related strokes are poor, with a 5-year survival rate of 39.2%, 5-year recurrence rate of 21.5%, and 25.9% of patients requiring a nursing home. 6 Cardioembolic stroke related to AF is a worrying public health concern, especially with population aging increasing its prevalence. Hence, the aim of the present study was to determine the frequency of cardioembolic stroke caused by AF, the previous diagnoses of the disease, and the previous treatment patterns in the s city of Joinville, state of Santa Catarina, southern Brazil.

## METHODS

A population-based study was performed in Joinville. The sample was collected from individuals who were residing in the city, whose population, according to the 2010 Brazilian Census, 7 was of 515,228 inhabitants; in 2015, the population was estimated at 562,151 inhabitants.

Data from January 2017 to December 2020 were extracted from the database of the municipal stroke notification program, called JOINVASC, which started in 2005. Methods for stroke assessment have been previously published. 8 In summary, we used multiple overlapping assessment sources according to the World Health Organization (WHO) STEPwise approach to noncommunicable disease (NCD) risk factor surveillance (STEPS) criteria, 8 therefore including every patient with a stroke in the city, as well as every hospital, ambulatory cases, and death certificates.

Demographic data, cardiovascular risk factors, electrocardiograms (ECGs), echocardiograms, carotid and transcranial Doppler, and laboratory tests were used to classify the stroke etiology. The diagnosis of stroke followed established criteria. 8 9 10 The etiology of the stroke was established according to the Trial of ORG 10127 in the Acute Stroke Treatment (TOAST) study. 11 The stroke investigation routine followed the guidelines of the Brazilian Society of Cerebrovascular Diseases. 12

Upon presentation of the free and informed consent form by one of the research nurses of the Joinville Stroke Registry, patients who refused to participate were excluded from the study.

The diagnosis of AF was divided into: “new AF,” “known AF,” “paroxysmal AF”, and “new and paroxysmal AF.” “New AF” was defined as AF confirmed in the admission report after stroke and sustained by more than one ECG. Patients with “known AF” were those who had already had more than one ECG with reported AF, which were confirmed upon admission through an ECG or Holter test. “Paroxysmal AF” was defined as AF in at least one previous report but not others. Finally, “new and paroxysmal AF” was defined when AF was confirmed in the admission report after stroke but not maintained.

Descriptive statistics and a binomial 95% confidence intervals (95%CIs) were used to calculate the percentages. The information was inserted into Excel (Microsoft Corp., Redmond, WA, US) spreadsheets and, to minimize errors, it was inserted in double entries. The results were compiled in absolute numbers and percentages, with two independent reviewers checking them.

Informed consent was obtained from all of the subject(s) and/or their guardian(s). The study was approved by the Ethics Committee of Hospital Municipal São José (under number 4.653.003).

## RESULTS

Between 2017 and 2020, there were 3,303 cases of ischemic stroke, 593 (18%) of which were cardioembolic. The crude incidence rate of ischemic stroke was calculated as 140.7 cases per 100 thousand inhabitants, and, for cardioembolic stroke, as 25.3 cases per 100 thousand inhabitants. Table 1 shows the population-based incidence of ischemic stroke and the cardioembolic subtype in the analyzed period.

**Table 1:** Incidence of ischemic and cardioembolic stroke in Joinville between 2017 and 2020

Year	Ischemic stroke		Cardioembolic stroke		Proportion of ischemic strokes (95% confidence interval)
	n	Cases per 100 thousand inhabitants	n	Cases per 100 thousand inhabitants	
2017	838	145.6	164	28.5	19.6% (16.9%–22.3%)
2018	800	137.2	147	25.2	18.4% (15.7%–21.1%)
2019	839	142.1	142	24.0	16.9% (14.4%–19.5%)
2020	826	138.2	140	23.4	16.9% (14.4%–19.5%)
Total	3,303	140.7	593	25.3	18.0% (16.6%–19.3%)

There were 360 patients with cardioembolic stroke related to AF (60.7% of cardioembolic strokes), and no patients with AF classified as another stroke subtype. Most patients (222; 61.6%), had known AF, and 94 (26.1%) were diagnosed with AF after the stroke ( Table 2 ). Most patients were aged between 70 and 89 years (229; 63.6%).

**Table 2:** Atrial fibrillation in patients with cardioembolic stroke between 2017 and 2020

		N	Proportion (95% confidence interval)
Previous diagnosis of atrial fibrillation	Newly-diagnosed atrial fibrillation	94	26.1% (21.6%–30.6%)
	Previously-known atrial fibrillation	222	61.7% (56.6%–66.7%)
	Previously-known paroxysmal atrial fibrillation	36	10.0% (6.9%–13.1%)
	Newly-diagnosed paroxysmal atrial fibrillation	8	2.2% (0.7%–3.7%)
Age in years of the patients with atrial fibrillation	0–39	2	0.6% (0.0%–1.3%)
	40–49	5	1.4% (0.2%–2.6%)
	50–59	28	7.8% (5.0%–10.5%)
	60–69	78	21.7% (17.4%–25.9%)
	70–79	114	31.7% (26.9%–36.5%)
	80–89	115	31.9% (27.1%–36.8%)
	≥ 90	18	5.0% (2.7%–7.3%)

In addition, Table 3 details the previous use of medication among patients with known or paroxysmal AF. Of all patients with AF, only 170 (47.2%) reported previous use of anticoagulants. Most patients (79.3%) reported regular use of medication, and 9.2% had stopped using their medication before the stroke. Of the anticoagulants, 35.9% used warfarin, 34.7%, direct oral anticoagulants (DOACs), and 29.4% did not have the type of anticoagulant registered.

**Table 3:** Previous medication use among patients with known or paroxysmal atrial fibrillation between 2017 and 2020

		N	Proportion (95% confidence interval)
Type of previous medication use among patients with atrial fibrillation	Anticoagulants	170	47.2% (42.1%–52.4%)
	Other medication	88	24.4% (20.0%–28.9%)
Anticoagulant used (among patients with reported use)	Warfarin	61	35.9% (28.7%–43.1%)
	Direct oral anticoagulants	59	34.7% (27.5%–41.9%)
	Not registered	50	29.4% (22.6%–36.3%)
Patterns of previous use of medication	Takes the medication regularly	146	79.3% (73.5%–85.2%)
	Takes the medication irregularly	21	11.4% (6.8%–16.0%)
	Stopped using the medication	17	9.2% (5.1%–13.4%)

## DISCUSSION

The present study provides evidence about the incidence of cardioembolic stroke, stroke related to AF, and previous use of anticoagulants among patients with AF in Brazil. Of the patients with stroke caused by AF, ∼ 71.6% had a previous AF diagnosis, and 47.2% were being treated with an anticoagulant. Regarding the previous use of anticoagulants, about half of the patients used DOACs.

Although patients with AF are more likely to experience an ischemic stroke, the risk is not uniform and varies depending on other risk factors, such as previous stroke, hypertension, advancing age, and diabetes. 13 Anticoagulation is recommended for patients at moderate or high risk of stroke, while patients at low risk are treated with aspirin or no antithrombotic medication. 14 15 Nonetheless, a systematic review 16 including 54 studies suggests widespread underuse of anticoagulants in patients at a high risk of stroke. This finding is consistent with those of our study, and it implies that many cases of stroke could be avoided by programs that either seek out patients who have AF but are undiagnosed or encourage the evidence-based prescription of anticoagulants for those who already have the diagnosis.

Amaral et al. 17 (2017) performed a study in Joinville between 2014 and 2015 with a similar objective. Between 2014 and 2015, 374 ischemic strokes were recorded, 84 (22.5%) of which were cardioembolic. The prevalence of cardioembolic stroke in our region was below the average reported by Hajat et al. 18 (2011) (27.8%) and O'Carroll and Barrett 19 (2017), of 20% to 30%.

Regarding the proportion of patients with known and unknown AF, in agreement with the study by Amaral et al. 17 (2017), there was a higher percentage prevalence of known AF. 17 In a study conducted in Germany, Rizos et al. 20 (2011) also evaluated the proportion of previously-known AF in patients with stroke or transient ischemic attack. They 20 demonstrated that a previously-documented history of AF was present in 19.7% of the patients, highlighting the fact that paroxysmal AF was more prevalent than persistent AF, which differs from the results of the present study. A study 21 of 112 patients admitted to a hospital in Buenos Aires, Argentina, discovered that 73 were not anticoagulated, with 61 (83.6%) of those patients having never received oral anticoagulation, and 12 (16.4%) having recently discontinued anticoagulation. Sposato et al. 22 (2015), through a meta-analysis of 28,290 studies, showed that 7.7% of stroke patients were diagnosed with AF in the emergency room after the acute event.

Furthermore, we highlight the possibility that the prevalence of AF was underestimated in the present study due to the existence of paroxysmal AF. In the present study, patients with suspected cardioembolic stroke performed a 24-hour Holter test. However, the test may be insufficient to diagnose paroxysmal AF. A study performed in Sweden showed a 49% increase when the AF diagnostic technique was changed from a 12-lead ECG to a portable ECG recording 20 to 30 seconds twice a day and if palpitations occurred. 3

Finally, as limitations of the present study, we emphasize the possibility of underestimated data due to undiagnosed paroxysmal AF, as well as statistical limitations due to the absence of assessment of confounding factors. Another limitation was the possibility of recall bias in relation to the previous diagnosis of AF and the use of medications for this condition.

In conclusion, during the analyzed period, cardioembolic stroke caused by AF led to a significant stroke burden in Joinville, and a large proportion of patients had undiagnosed AF or previously-diagnosed AF not treated with anticoagulants.
